# O.5.3-3 Evaluation of the Sports Club for Health (SCforH) online learning tool among various stakeholders and end-users from 34 European countries

**DOI:** 10.1093/eurpub/ckad133.254

**Published:** 2023-09-11

**Authors:** Tena Matolić, Danijel Jurakić

**Affiliations:** University of Zagreb, Faculty of Kinesiology, Croatia; tena.matolic@kif.hr; University of Zagreb, Faculty of Kinesiology, Croatia; tena.matolic@kif.hr

## Abstract

**Purpose:**

The awareness of the Sports Club for Health (SCforH) guidelines was shown to be positively associated with a greater commitment to HEPA promotion in sports organisations. Because of the low implementation (<20%) of SCforH guidelines, we created their e-version, an interactive multilingual online learning course. The aim of this research was to determine the levels of potential stakeholders’ perceived quality of the course, to help improve the: i) content of new, similar courses, ii) effectiveness of their future dissemination, and iii) the rate of their success.

**Methods:**

The sample included 910 participants: 26 HEPA policymakers, 15 HEPA promotors and researchers, 55 sports associations, and 219 sports club representatives, 70 academic staff, and 410 students in higher education or research institution in the field of sport, health, or physical activity, and 115 “other” participants, from 34 European countries. The participants provided their answers to the EDUCA-TOOL course evaluation questionnaire consisting of 12 course-quality assessment items categorised into 4 different subscales: Reaction, Learning, Behavioural intent, and Expected outcomes. The summary scores for all subscales (max = 25) and the total score (max = 100) were calculated.

**Results:**

Overall reaction and learning mean were 21.11 and 19.63, ranging from 20.58 to 22.33 for reaction, and from 18.81 to 20.94 for learning among students, and HEPA promotors and researchers, respectively. The overall behavioural intent mean was 20.12, ranging from 19.29 in students to 21.68 in HEPA policymakers. The expected outcomes mean was 19.31, ranging from 18.39 in academic staff to 21.7 in HEPA promotors and researchers. Overall satisfaction with the course was relatively high – 80.43 mean, and in a range from 77.72 for students to 86.51 in HEPA promotors and researchers.

**Conclusions:**

Innovative and engaging methods of disseminating the SCforH guidelines may aid their widespread adoption and understanding among various stakeholders in Europe. These findings can also be used to improve the content of new training courses for HEPA promotion.

**Support/Funding Source:**

This work is supported by the Erasmus+ Program of the European Union as part of the project “Creating Mechanisms for Continuous Implementation of the Sports Club for Health Guidelines in the European Union” under Grant: 613434-EPP-1-2019-1-HR-SPO-SCP.

